# CAM Use in Pediatric Neurology: An Exploration of Concurrent Use with Conventional Medicine

**DOI:** 10.1371/journal.pone.0094078

**Published:** 2014-04-15

**Authors:** Elaine Galicia-Connolly, Denise Adams, Justin Bateman, Simon Dagenais, Tammy Clifford, Lola Baydala, W. James King, Sunita Vohra

**Affiliations:** 1 CARE Program, Department of Pediatrics, University of Alberta, Edmonton, Alberta, Canada; 2 Faculty of Medicine & Dentistry, University of Alberta, Edmonton, Alberta, Canada; 3 Palladian Health, West Seneca, New York, United States of America; 4 Departments of Pediatrics and of Epidemiology & Community Medicine, University of Ottawa, Ottawa, Ontario, Canada; Canadian Agency for Drugs and Technologies in Health, Ottawa, Ontario, Canada; Department of Pediatrics, Faculty of Medicine and Dentistry, University of Alberta, Edmonton, Alberta, Canada; 6 Division of Pediatric Medicine, Department of Pediatrics, University of Ottawa, Ottawa, Ontario, Canada; Children's Hospital of Eastern Ontario, Ottawa, Ontario, Canada; 7 CARE Program, PedCAM Network, Department of Pediatrics, Faculty of Medicine & Dentistry and School of Public Health, University of Alberta, Edmonton, Alberta, Canada; Sudbury Regional Hospital, Canada

## Abstract

**Background:**

Previous studies have found that up to 60% of children with neurologic conditions have tried complementary and alternative medicine (CAM).

**Objective:**

To assess the use of CAM among patients presenting to neurology clinics at two academic centers in Canada.

**Methods:**

A survey instrument was developed to inquire about use of CAM products and therapies, including reasons for use, perceived helpfulness, and concurrent use with conventional medicine, and administered to patients or their parents/guardians at the Stollery Children's Hospital in Edmonton and the Children's Hospital of Eastern Ontario (CHEO) in Ottawa.

**Results:**

Overall CAM use at the Stollery was 78%, compared to 48% at CHEO. The most common CAM products used were multi-vitamins (84%), vitamin C (37%), homeopathic remedies (24%), and fish oil/omega 3 s (22%). The most common CAM practices used were massage (47%), chiropractic (37%), faith healing (18%), aromatherapy (16%), homeopathy (16%), and relaxation (16%). Many patients used CAM products at the same time as conventional medicine but just over half (57%) discussed this concurrent use with their physician.

**Conclusion:**

CAM use is common in pediatric neurology patients and most respondents felt that it was helpful, with few or no harms associated. However, this use is often undisclosed, increasing possibility of interactions with conventional drugs. We urge clinicians to inquire about CAM use during routine history taking at every patient visit. Parents would clearly like more information about CAM from their specialty clinics; such information would be easier to share if more primary data were available about the safety and effectiveness of commonly used therapies.

## Introduction

The use of complementary and alternative medicine (CAM) is common among children, particularly those with chronic, recurrent or incurable conditions [1]. For instance, a study in 2001 found that 62% of chronically ill children and adolescents attending subspecialty clinics in Salt Lake City (n = 505) had used dietary supplements [2]. Another study in 2003 found that 64% of children (n = 141) attending a rheumatology clinic in Toronto were using CAM at the time, with 50% using more than one type of CAM [3]. Among pediatric oncology patients, CAM use increased from 16% in 1977 to over 65% in the late 1990s [4]–[7].

CAM use has been reported for a variety of neurologic conditions, including epilepsy, headaches, traumatic brain injury, neuromuscular disorders, developmental delay, and degenerative brain diseases [8]–[14]. A study released in 2003 found that 14% of children with epilepsy attending a neuropediatric unit at an Israeli hospital were currently using CAM (n = 115), while nearly one-third had used CAM in the past [15]. A survey of 17 Chinese-Canadian children with stroke and cerebrovascular disease in Toronto found that most (53%) had used Chinese herbs [16]. In 2005, a study at the Alberta Children's Hospital neurology clinic in Calgary showed that 44% of patients used CAM, with many of those patients receiving more than one type of CAM [8].

The purpose of this study was to assess the use of CAM among pediatric neurology patients and explore beliefs and attitudes towards CAM, including perceived helpfulness, sources of and trust in CAM information sources, concurrent use with conventional drugs, disclosure of CAM use to health care providers and adverse events.

## Methods

This paper is part of a larger study that was carried out at two sites in Canada: the Stollery Children's Hospital (Stollery) in Edmonton, Alberta and the Children's Hospital of Eastern Ontario (CHEO) in Ottawa, Ontario. Five pediatric clinics were chosen for the larger study as follows: cardiology, gastrointestinal, neurology, oncology, and respiratory. These five subspecialties were chosen because many patients with chronic conditions who may be likely to use CAM products and/or therapies attend them. Participants (children and/or their families) were eligible to take part in this study if they could read French or English, were under 18 years of age, and had not previously filled out a questionnaire for this study. Full methods may be found in Adams 2013 [17]. Ethical approval was granted by the CHEO and Stollery Research Ethics Boards.

Data was entered into an SPSS database (version 11). Descriptive statistics were presented as means (standard deviation) or medians (IQR) for continuous variables and as numbers and percentages for categorical variables. Demographics, health status, use of specific CAM therapies and products, satisfaction with care and beliefs about CAM were compared by centre (Stollery vs. CHEO) using Wilcoxon tests, independent t-tests and chi-square tests. CAM use was compared between centres and modeled through logistic regression (univariate and multivariate models). Predictive variables included child's age, child's gender, child's health status, time since diagnosis, ethnicity, parental education and income, family's use of CAM, family's CAM insurance, and discussion of CAM with conventional medical practitioners. Regression diagnostics such as c-statistics, r-squared and Hosmer and Leme show lack-of-fit statistics were performed and measures for detecting influential observations and outliers were also considered.

## Results

### Population Characteristics

A total of 206 surveys were completed, including 151 (73.3%) at Stollery and 55 (26.7%) at CHEO, with an equal proportion of male and female patients, and a mean age of 9.6 years. Patient ethnicity was self-identified as Caucasian/Canadian/French Canadian (86.7%), First Nations/Inuit/Metis (9.7%), South Asian (4.6%), Black (4.1%), East Asian (2.0%), Middle Eastern/Arabic (1.0%), or Latin American/Hispanic (0.5%). Most patients reported their health status as good to excellent (92.7%) and had received their diagnosis more than 12 months ago (62.3%). The most common diagnosis was epilepsy (50.5%), followed by headaches/migraines (9.2%) and cerebral palsy (4.9%). More than three-quarters (77.5%) of Stollery patients had previously used CAM, compared to half (48.1%) at CHEO (p<0.0001) (Table 1).

**Table 1:** Demographic Information.

	n	Edmonton	n	Ottawa	Total
**Patient Information**					
Child/Youth Age mean (SD)	151	9.3 (4.9)	54	10.3 (4.6)	9.6 (4.8)
*Gender* Female N (%)	151	78 (51.7)	54	31 (57.4)	109 (53.2)
*Time since diagnosis*	146		53		
0–3 mo* o>e 0.0214		11 (7.5)		10 (18.9)	21 (10.6)
3–6 mo		13 (8.9)		5 (9.4)	18 (9.0)
6–12 mo		28 (19.2)		8 (15.1)	36 (18.1)
more than 12 mo		94 (64.4)		30 (56.6)	124 (62.3)
*If child/youth has ever used Cam* Yes*e>o <0.0001	151	117 (77.5)	54	26 (48.1)	143 (69.8)
**Parent/Caregiver Information**					
Age mean (SD)	146	39.4 (7.6)	51	39 (7.5)	39.3 (7.6)
*Gender* Female N (%)	149	133 (89.3)	54	49 (90.7)	182 (89.7)
*Highest completed level of education*	145		53		
No formal education		1 (0.7)		0	1 (0.5)
Primary school only		1 (0.7)		2 (3.8)	3 (1.5)
Secondary school		31 (21.4)		12 (22.6)	43 (21.7)
Registered apprentice or other trade		6 (4.1)		2 (3.8)	8 (4.0)
College, CEGEP, or other non-university		53 (36.6)		14 (26.4)	67 (33.8)
University, without a university degree		14 (9.7)		2 (3.8)	16 (8.1)
University, with a university degree		32 (22.1)		21 (39.6)	53 (26.8)
Other		7 (4.8)		0	7 (3.5)
*Annual household income*	142		50		
Less than $10,000		5 (3.5)		0	5 (2.6)
$10, 000–$19,999		6 (4.2)		2 (4.0)	8 (4.2)
$20,000–$39,999		17 (12.0)		8 (16.0)	25 (13.0)
$40,000–$79,999		48 (33.8)		18 (36.0)	66 (34.4)
$80,000 and over		66 (46.5)		22 (44.0)	88 (45.8)
*If respondent had ever used CAM* Yes*e>o 0.0455	149	110 (73.8)	54	32 (59.3)	142 (70.0)

*- multiple responses allowed.

n - number with valid response.

Parents/caregivers in attendance at the clinic had a mean age of 39.3 years and were mostly mothers (84.2%). Over 70% reported attending post-secondary education and 80.2% reported a household income of $40,000 or more. Most (94%) respondents reported that they were knowledgeable about the child's use of CAM. Among the parents/caregivers, 73.8% of those at the Stollery had used CAM before, compared to 59.3% at CHEO (p = 0.0455). Use of CAM by Stollery patients was found to be 21 times higher when parents/caregivers also used CAM (95% confidence interval (CI): 7.6–58.2, p<0.0001); no such association was found for CHEO patients.

### Products/Practices

The most common CAM products (also known as natural health products) ever used were multi-vitamins (89.9%), vitamin C (36.7%), cold remedies (23.7%), teething remedies (21.6%), ear drops (17.3%), and fish oils/omega 3 s (21.6%). Patients whose CAM use was limited to multivitamins/minerals accounted for only 11% of respondents (14% at Stollery and 0% at CHEO, p<0.0001) The most common CAM practices ever used were massage (47.1%), chiropractic (36.8%), faith healing (18.4%), aromatherapy (16.1%), and relaxation (16.1%). Most products and practices were reported to be helpful. More details on previous and current CAM use, as well as their perceived helpfulness, is presented in Table 2. Common reasons for not using CAM reported for both caregivers and children included not knowing enough about CAM, not believing it to be necessary for them, and worry about side effects from combing CAM with conventional care.

**Table 2:** Commonly Used Products/Practices and their Perceived Helpfulness.

Product	Ever Used	Current Use	Perceived Helpfulness No. (%)	
	(n = 139) *No. (%)*	*(n = 73) No. (%)*		
			n	Yes *No. (%)*
*Vitamins and Minerals*	125 (89.9)	63 (86.3)		
Multi-vitamin	116 (83.5)	56 (76.7)	105	52 (49.5)
Folic Acid	12 (8.6)	7 (9.6)	10	6 (60.0)
Vitamin B	18 (12.9)	8 (11)	15	6 (40.0)
Vitamin C	51 (36.7)	18 (24.7)	43	27 (62.8)
Calcium	22 (15.8)	11 (15.1)	19	9 (47.4)
*Herbals*	35 (25.2)	13 (17.8)		
Echinacea	22 (15.8)	3 (4.1)	21	13 (61.9)
Goldenseal	9 (6.5)	6 (8.2)	8	4 (50.0)
Peppermint	11 (7.9)	4 (5.5)	9	7 (77.8)
*Homeopathics*	53 (38.1)	9 (12.3)		
Cold remedy	33 (23.7)	7 (9.6)	29	24 (82.8)
Colic remedy	16 (11.5)	1 (1.4)	13	8 (61.5)
Ear drops	24 (17.3)	2 (2.7)	17	14 (82.4)
Teething remedy	30 (21.6)	0	23	20 (87.0)
*Miscellaneous*	47 (33.8)	22 (30.1)		
Acidophilus/probiotics	14 (10.1)	7 (9.6)	12	5 (41.7)
Fish oil/omega 3 s *	30 (21.6)	14 (19.2)	26	11 (42.3)
Flax oil	11 (7.9)	2 (2.7)	9	8 (88.9)
Other **	19 (13.7)	10 (13.7)	NA	NA
**Practice**	N = 87	N = 37		
Aboriginal Healing	8 (9.2)	3 (8.1)	7	7 (100)
Acupuncture	8 (9.2)	1 (2.7)	7	4 (57.1)
Aromatherapy	14 (16.1)	4 (10.8)	13	7 (53.8)
Chiropractic	32 (36.8)	6 (16.2)	32	23 (71.9)
Faith healing	16 (18.4)	7 (18.9)	13	12 (92.3)
Homeopathy	14 (16.1)	2 (5.4)	11	9 (81.8)
Magnets	6 (6.9)	1 (2.7)	5	2 (40.0)
Massage	41 (47.1)	13 (35.1)	35	28 (80.0)
Naturopathy	9 (10.3)	1 (2.7)	9	7 (77.8)
Relaxation	14 (16.1)	8 (21.6)	10	8 (80.0)
Yoga	8 (9.2)	3 (8.1)	8	2 (100)

*use of fish oil ever at CHEO (40.0%) was significantly more than at the Stollery (17.5%), p = 0.0134.

**neutralizer to vaccine, yogurt capsule, wheat germ, flax, Q10, cranberry, Manatech, melatonin, sea salt, fiber.

### Safety Issues

Only 8.7% of respondents used CAM before trying conventional medicine, while 46.1% reported concurrent use of CAM and conventional medicine. Children at CHEO reported significantly more concurrent use of CAM (66.7%) than those at Stollery (41.5%) (p = 0.03). Of the 77 respondents who reported concurrent use of CAM with prescription drugs, 74 (96%) listed the types of drugs and CAM products or practices involved (Table 3). The most common combination reported was concurrent use of anticonvulsant drugs and vitamins/minerals (n = 34), followed by anticonvulsant drugs and herbal products (n = 7), and psychostimulants and vitamins/minerals (n = 6).

**Table 3:** Conventional and CAM concurrent use.

Therapeutic agent	CAM products used concurrently
Anticonvulsant agents*	Vitamins and minerals
	Herbals
	Homeopathics
	Probiotics
	Fish oil/Omega 3
Psychostimulants (i.e. Cocerta, Ritalin, Dexedrine, Strattera)	Vitamins and minerals
	Herbals
	Probiotics
	Fish oil/Omega 3
Antibiotics	Vitamins and minerals
	Probiotics
Antipsychotics (i.e. risperdal)	Vitamins and minerals
	Herbals
	Probiotics
	Fish oil/Omega 3
Antidepressants	Vitamins and minerals
	Herbals
	Fish oil/Omega 3
Others**	Vitamins and minerals
	Probiotics

*Includes seizure medications;

**Antacids or Antiemetics (4), Diuretics (1), Inhalers (5).

Twenty-one side effects were reported, with most being minor (i.e. self-resolving; not needing medical care). However, five side effects of moderate severity (i.e. medical care was sought) were also reported, one each for multi-vitamins, Echinacea, teething remedy, aromatherapy, and yoga. Only two severe side effects were reported, associated with multi-vitamins (n = 1) and magnets (n = 1). No further details were provided by respondents.

The most commonly used sources of information about CAM included families (63.7%), pharmacies (44.4%), books (38.7%), and the Internet (37.1%). The most trusted sources of information about CAM (on a 0–10 scale) were conventional health care providers (8.4), pharmacies (8.2), CAM providers (8.1), and their neurology clinic (7.9). Health food stores were perceived as more trustworthy sources of information at CHEO (8.8) than at the Stollery (6.7) (p = 0.03).

Among those who reported using CAM and conventional drugs concurrently, 56.8% consulted their medical doctor about this use, and 43.5% reported consulted their pharmacist. Although a large majority (81.1%) of respondents agreed or strongly agreed that they felt comfortable discussing CAM at the neurology clinic, most (66.3%) reported that they would like more information on CAM use at the neurology clinic, and would be more likely to use CAM products (61.3%) and practices (64.3%) if they were made available in the neurology clinic.

## Discussion

This study details the prevalence and patterns of use of CAM in pediatric neurology clinics at two academic institutions. Use between our two sites was significantly different (74% vs 59%) and statistical modeling showed that the only variable that influenced child CAM use was parent CAM use; however this was only evident at the Stollery and it is likely that our small sample size at CHEO did not allow us to detect influence at that site. Other variables that approached significance were child age, parent education and CAM insurance status.

A study based on the US National Health statistics in 2007 reported that CAM use among children was five times higher when the parent used CAM. Other factors associated with CAM use were higher parent education and income, access to private health insurance, worries over cost delayed receipt of conventional care and the inability to afford conventional medical care [18], [19]. Our study supports the importance of these variables in predicting child CAM use.

Multivitamins/nutritional supplements, massage, and chiropractic care were the most commonly used CAM. Recent evidence supports the use of some types of CAM for neurologic conditions [20] such as biofeedback or neurofeedback for chronic pediatric headaches [21] and fish oil supplements for ADHD [22]. However, evidence for other types of CAM is limited, including for vitamins [23], ketogenic diet [24], acupuncture [25] or yoga [26]. CAM approaches warrant further study, as patients and families are interested in alternatives to conventional pharmacotherapy.

Similar to Soo et al. [8], most of our respondents perceived that CAM products or therapies were generally helpful, with few moderate or severe side effects, but only half discussed their child's CAM use with their physician.

Novel information that our study provides includes an exploration of concurrent use of CAM and conventional medicine, including rates and patterns of use as well as safety and related disclosure to health care providers. We also investigated issues of such as source and trust in CAM info sources as well as information needs of participants in relation to their neurology clinic.

Concurrent use of CAM and prescription medications was common, which may be attributed to comorbidities among patients with neurological conditions such as epilepsy, migraines, and ADHD; the side effects common to many pharmacologic therapies for these conditions; and their somewhat chronic or refractory nature [27]. Just over half of our population was diagnosed with epilepsy. Epilepsy patients who concomitantly use herbs, vitamins and minerals with their antiepileptic drugs (AED) are at potential risk of drug interactions or serious adverse events. For example, herbs and supplements (i.e. Echinaceae, evening primrose oil, gingko biloba), are known to decrease seizure threshold [28], [29], while vitamins can have a direct convulsant effect (folic acid), alter drug metabolism (niacin, pyridoxine), or reverse the effects of antiseizure medications to worsen seizure control (folic acid) [30].

Although health care providers were considered the most trusted source for CAM information, over 40% of patients did not discuss concurrent drug-CAM use with their physicians. This finding is of concern and provides an opportunity to recommend updating medical training to not only elicit CAM use, but more importantly, to equip future physicians to confidently address the efficacy and safety of CAM in their patients. Patients may also feel more inclined to be open with their physicians if CAM use was integrated with a patient's medication history. We recommend that clinicians routinely inquire about CAM use during each visit, keeping in mind that some patients may fail to disclose this information for fear of being reprimanded by their physician. Open honest communication between patient and physician is more likely when physicians do not have a judgmental attitude regarding their patients' CAM use [31]. Useful resources for clinicians regarding the safety and efficacy of pediatric CAM include the American Academy of Pediatrics *Pediatrics in Review* CAM series (Headaches PIR Feb 2010, Fish oils and Neurodevelopmental disorders PIR Apr 2009, Learning Disabilities Feb 2011), the rapid tool for checking for herbal and drug interaction [32], subscription databases, such as Natural Standard and Natural Medicines Comprehensive Database, and open access websites, such as NCCAM (www.nih.nccam.gov) and PedCAM (www.pedcam.ca).

Limitations of this study include those of recall of events long past, which may be exacerbated by our use of a parent proxy, however, parents are routinely asked about aspects of their child's health and often, this discourse occurs during an annual medical check-up. In addition, recent evidence suggests that recall of regularly consumed natural health products, measured by a single questionnaire, is comparable to more detailed methods such as use of a diary [33]. Our choice of a period prevalence less than a full calendar year could result in estimates of CAM use that are confounded by the season during which the survey was completed. Because CAM use is known to vary between ethnic groups [9], [34]–[37], administration of our questionnaires in English or French only may limit the generalizability of our findings.

## Conclusions

CAM use is common in pediatric neurology patients and most respondents felt that it was helpful, with few or no associated harms. However CAM use is often undisclosed to physicians and pharmacists, increasing the possibility of interactions with conventional drugs – we urge clinicians to inquire about CAM use during routine history taking at every patient visit. Parents would clearly like more information about CAM from their specialty clinics; such information would be easier to share if more primary data were available about the safety and effectiveness of commonly used therapies.
